# Dissociative Amnesia Complicating an Attempted Maternal Filicide-Suicide: A Case Report

**DOI:** 10.7759/cureus.98753

**Published:** 2025-12-08

**Authors:** Wei Aun Goh, Shiao Ling Ling

**Affiliations:** 1 Department of Psychiatry, Hospital Tengku Permaisuri Norashikin, Kajang, MYS

**Keywords:** dissociation, dissociative amnesia, filicide-suicide, homicide-suicide, maternal filicide, murder-suicide, parental psychopathology

## Abstract

Filicide-suicide, a subset of homicide-suicide, describes cases in which one or both parents kill their child(ren) and subsequently die by suicide. It is a rare but tragic phenomenon with limited reports in Malaysia. Dissociative amnesia, characterized by an inability to recall important autobiographical information, often related to a traumatic experience, can further complicate this phenomenon. We present a case of a young woman who inflicted life-threatening injuries on her toddler and herself, and subsequently exhibited features of dissociative amnesia after she regained consciousness. One month later, her recovery was accompanied by intense emotional distress and guilt. This case highlights the diagnostic complexity of dissociative amnesia in the aftermath of severe trauma and the need for individualized management in cases involving attempted filicide and suicide.

## Introduction

Dissociative amnesia is a psychiatric condition characterized by an inability to recall important autobiographical information, often related to traumatic or highly stressful events [1]. The memory loss in dissociative amnesia is inconsistent with ordinary forgetting and cannot be explained by medical conditions or substance use [1]. Individuals with dissociative amnesia often experience sudden and selective amnesia, particularly for distressing events, despite intact cognitive function. This condition is frequently associated with acute psychological distress and may emerge as a response to overwhelming emotional trauma in the psychological stress model hypothesis [2].

Filicide, the act of a parent killing their child, is a rare but profoundly disturbing event with complex psychiatric underpinnings [3]. When accompanied by a suicide attempt, filicide-suicide cases often involve significant psychopathology with high rates of mental illness [4]. Although fewer studies describe filicide-suicide as a discrete category, literature on filicide consistently shows an overrepresentation of mental illness, including affective disorders, psychotic disorders, and personality disorders [5]. However, the presence of dissociative amnesia in the aftermath of a filicide-suicide attempt is rarely documented and remains an underexplored area in psychiatric literature. Interference with memory processing can occur following extreme psychological distress, leading to profound memory fragmentation and a genuine dissociative state as a result [6].

In this report, we describe a case of a young woman with no prior psychiatric history who attempted filicide-suicide, followed by an episode of dissociative amnesia, in which she was unable to recall the event for over a month. As memory gradually returned through intrusive nightmares and partial recollections, she displayed significant emotional distress and depressive symptoms. This case highlights the diagnostic and management challenges of dissociative amnesia following extreme psychological trauma and underscores the importance of a comprehensive psychiatric approach in cases where filicide occurs without apparent psychosis or a clear motivational trigger.

## Case presentation

This is a young, married woman in her early 20s and a first-time mother to an 18-month-old boy, with no prior psychiatric history. She was found unconscious at home and had a laceration on her neck and multiple stab wounds to her abdomen. Her 18-month-old son was found beside her in critical condition with similar injuries, including a neck laceration and multiple abdominal stab wounds. Footage from inside the house recorded the alleged event and the sequence of how the injuries were inflicted. The clinical team reviewed the footage. She was seen carrying a knife from the kitchen and then walking into the living room while holding her son. It was observed that her son had already sustained a laceration to the neck at that time. She subsequently knelt on the floor, appeared visibly distressed and tearful, and made a phone call to her husband to apologize. She then pinned her child to the ground and proceeded to stab his abdomen multiple times, displaying a blunted affect. Despite his severe injuries, the child attempted to crawl away; however, she followed him and continued stabbing his abdomen. She then turned the weapon on herself, inflicting multiple stab wounds to her abdomen and slashing her neck several times before eventually losing consciousness. She was transported to the emergency department by ambulance, while her son was taken to the Women and Children’s Centre for further management.

The collateral history obtained revealed that approximately two weeks before the incident she experienced a depressed mood and feelings of hopelessness. However, she did not disclose any concrete plan for suicide or homicide. Prior to the incident, she had a good bond with her son. Both she and her husband worked during the daytime, and the child was cared for by a babysitter when they were away at work. After work, they would pick the child up and spend the evening together. On the day of the incident, she was not on duty and was therefore taking care of her child alone at home. Two weeks before the incident, her mother observed that she appeared increasingly overwhelmed by her caregiving responsibilities, particularly in caring for her young son and handling his frequent tantrums. Neither her husband nor her mother observed any other behavioral changes or symptoms of mood instability, neglect, psychosis, or any previous dissociative episode. Her main support was her spouse. There were no issues in the marital relationship, nor were there any known stressors related to finances or interpersonal relationships, as reported by the patient and her family. There was also no history of childhood abuse or dissociative episodes in the past.

Emergency and surgical management

Upon arrival at the emergency department, the patient was in hypovolemic shock with a reduced Glasgow Coma Scale (GCS) score. Examination revealed a superficial laceration on her neck and multiple deep stab wounds to her abdomen. Despite fluid resuscitation, she remained hemodynamically unstable and required emergency exploratory laparotomy. Significant intraoperative findings included stomach perforations and multiple injuries to the abdominal wall and surrounding anatomical structures. She was stabilized in the ICU postoperatively before being transferred to the surgical ward and subsequently to the psychiatric ward for further evaluation after her general condition had stabilized.

Meanwhile, the 18-month-old boy was brought to the Women and Children’s Centre in critical condition. He sustained multiple penetrating abdominal injuries and a laceration across the left side of his neck. He underwent emergency exploratory laparotomy, during which segmental resection of the transverse colon with primary anastomosis and primary repair were performed. His recovery was complicated by necrotizing fasciitis of the bilateral flanks and an extended-spectrum beta-lactamase (ESBL) *Escherichia coli* infection. He required multiple sessions of wound debridement, a transverse colostomy, and skin grafting. Despite the severity of his injuries, the child showed gradual clinical improvement and was eventually discharged in stable condition after two months of hospitalization. He was cared for by his father and babysitter upon discharge. The case was referred to the Department of Social Welfare for child protection assessment and follow-up.

Psychiatric assessment of patient

Upon initial psychiatric evaluation in the ICU, she showed a reduced emotional response, presenting an unreactive demeanor that appeared incongruent with the reported traumatic incident. She displayed a notably blunted affect with minimal variation in facial expression and monotonous, minimal speech. There was a notable memory gap, as she was unable to recall the incident leading up to her admission. She reported that she had been feeding her child before losing consciousness. She firmly denied suicidal or homicidal thoughts. There were no overt psychotic symptoms. Throughout her stay in the ICU and surgical ward, she appeared emotionally numb, displaying inappropriately minimal distress.

Over her hospital stay, she became more cooperative and forthcoming. However, she continued to deny active suicidal or homicidal intent and persistently failed to recall any details of the incident. The diagnosis of dissociative amnesia was made based on the Structured Clinical Interview for DSM-5. Her intent at the time of the incident remained unclear due to the selective amnesia, significantly limiting the ability to take a comprehensive history. There were no perceptual disturbances noted throughout her hospital stay.

The primary treating team engaged the neuropsychiatry and forensic psychiatry teams to facilitate a comprehensive assessment and guide appropriate management. During the patient’s psychiatric inpatient stay, she underwent screening for autoimmune disorders, epilepsy, and post-traumatic stress disorder (PTSD), all of which returned negative results. Although her family declined a lumbar puncture, EEG and neuroimaging, including CT and MRI of the brain, were performed as alternative investigations.

Recovery of memory

She began to regain her memories about a month into her hospital stay. These manifested as distressing nightmares depicting the bloody scene, followed by fragmented recollections. Eventually, she was able to recall most details of the incident with clarity. Her recovered memories included detailed descriptions of the moments leading up to the act, such as feeding her child, being overwhelmed by his persistent crying, and an overwhelming sense of hopelessness. She recalled acting on impulse, stabbing her son first, then experiencing panic and a sense of irreversible damage, which culminated in her own suicide attempt. However, she denied psychotic symptoms, dissociative states, or delusions of control at the time of the act. She rejected the notion of being driven by external commands or psychotic ideation.

The return of her memory was accompanied by profound guilt, sadness, and emotional breakdowns. These emotions were consistent with her reported sense of responsibility and remorse, further supporting the authenticity of the recovered memory rather than confabulated or externally suggested content. Upon further inquiry, she retrospectively described experiencing depressive symptoms for two weeks before the incident, including low mood, anhedonia, early-morning awakening, lethargy, feelings of worthlessness, and suicidal thoughts. She expressed feeling overwhelmed and emotionally disconnected leading up to the act. Her depressive episode preceded the incident, affecting her work performance and impairing her ability to cope with caregiving responsibilities. She did not disclose her symptoms to her husband and instead internalized her feelings. During the critical period on the ward, she was monitored closely for any suicidal behavior, dissociative symptoms, PTSD, and psychotic symptoms; none of these were observed.

Investigations

Neuroimaging with CT and MRI of the brain revealed no acute intracranial pathology or structural abnormalities. Neurophysiological study using EEG demonstrated normal sleep and wake brain waveforms without epileptiform discharges or focal abnormalities.

As shown in Table 1, initial laboratory investigations showed mild anemia and leucocytosis, likely attributable to perioperative blood loss and postoperative inflammatory response following the self-inflicted stab injury and subsequent surgical repair. These parameters normalized on serial monitoring, and no other significant hematological or biochemical abnormalities were noted. Autoimmune screening, including antinuclear antibody (ANA), complement levels, ESR, and CRP, showed no significant abnormalities. Serological tests for HIV, syphilis, hepatitis B surface antigen (HBsAg), and hepatitis C virus antibody (anti-HCV) were all non-reactive. Serum folate and vitamin B12 levels were within normal limits. Urine drug screening and a urine pregnancy test were both negative.

**Table 1:** Serial laboratory investigations.

Investigation	February 7, 2025	February 8, 2025	February 9, 2025	February 10, 2025	February 18, 2025	February 19, 2025	February 20, 2025
Haemoglobin (13.5-17.4 g/dL)	12.1	11.7	10.9	10.5	11.7	12.8	-
WBCs (4-11 ×10^9/L)	10.77	13.76	16.9	14.64	7.33	5.18	-
Platelets (142-350 ×10^3/µL)	272	209	211	245	627	463	-
Sodium (136-146 mmol/L)	136.3	131.3	134.8	136.3	137.3	-	-
Potassium (3.4-4.5 mmol/L)	3.47	3.7	3.9	4.09	3.95	-	-
Chloride (101-109 mmol/L)	105.7	105.6	108	109.4	103.1	-	-
Urea (2.8-7.2 mmol/L)	3.63	2.97	3.17	4.65	4.23	-	-
Creatinine (64-104 µmol/L)	73.2	52.7	50.5	47.1	62.5	-	-
Calcium (2.20-2.65 mmol/L)	-	2.36	2.31	2.32	2.59	-	-
Phosphate (0.81-1.45 mmol/L)	-	0.65	0.81	0.86	1.21	-	-
Magnesium (0.77-1.03 mmol/L)	-	0.6	0.76	0.78	0.82	-	-
Total Bilirubin (5.0-21.0 µmol/L)	13.64	37.53	13.77	9.7	10.94	-	-
Albumin (35-52 g/L)	40.5	32.4	31.1	31.2	42.7	-	-
Alanine Aminotransferase (ALT) (<50 U/L)	8.6	37.6	32	26.5	17.4	-	-
Alkaline Phosphatase (ALP) (43-115 U/L)	62.4	40.4	37.5	37.5	65.2	-	-
Aspartate Aminotransferase (AST) (<50 U/L)	16.3	53.3	-	33.1	19.7	-	-
Thyroid-stimulating Hormone (TSH) (0.38-5.33 µIU/mL)	-	-	-	-	1.615	-	-
Free Thyroxine (FT4) (7.86-14.41 pmol/L)	-	-	-	-	13.6	-	-
Prothrombin Time (PT) (11.7-15.3 seconds)	14.3	16.9	19.1	16.3	-	-	-
Activated Partial Thromboplastin Time (APTT) (30.3-46.5 seconds)	31.4	34.1	40.5	41.4	-	-	-
International Normalised Ratio (INR) (0.86-1.14)	1.07	1.27	1.45	1.23	-	-	-
CRP (<5)	-	-	-	-	-	-	1.632
ESR (<20)	-	-	-	-	-	-	54
Antinuclear Antibody (ANA)	-	-	-	-	-	-	Negative
Complement C3 (0.9-1.8 g/L)	-	-	-	-	-	-	1.353
Complement C4 (0.1-0.4 g/L)	-	-	-	-	-	-	0.342
Folate (7.02-45.09 nmol/L)	-	-	-	-	21.38	-	-
Vitamin B12 (180-914 pg/mL)	-	-	-	-	664	-	-
Rheumatoid Factor	-	-	-	-	-	-	Negative
HIV Antigen and Antibodies	-	-	-	-	-	Non-reactive	-
Rapid Plasma Reagin (RPR)	-	-	-	-	-	Non-reactive	-
Hepatitis B Surface Antigen (HBsAg)	-	-	-	-	-	Non-reactive	-
Hepatitis C Virus Antibody (Anti-HCV)	-	-	-	-	-	Non-reactive	-
Urine Drug Test	Negative	-	-	-	-	-	-
Urine Pregnancy Test	Negative	-	-	-	-	-	-

Psychological assessments are summarized in Table 2. They demonstrated intact cognitive functioning, with a Mini-Mental State Examination (MMSE) score of 30 and a Montreal Cognitive Assessment (MoCA) score of 26. Psychological symptom assessment indicated minimal to mild emotional distress. A Beck Depression Inventory (BDI) score of 12 suggested minimal depressive symptoms. A Beck Anxiety Inventory (BAI) score of 9 indicated low anxiety levels. The PTSD Checklist for DSM-5 (PCL-5) score was 24, which is below the usual clinical cutoff, suggesting that PTSD was unlikely, although some subthreshold symptoms may have been present. Cognitive-behavioural evaluation using the Automatic Thoughts Questionnaire (ATQ) yielded a score of 19, indicating a low frequency of negative automatic thoughts, consistent with a generally adaptive cognitive pattern.

**Table 2:** Psychological assessment.

Assessment Tool	Date	Score
Mini-Mental State Examination (MMSE)	February 20, 2025	30/30
Montreal Cognitive Assessment (MoCA)	March 4, 2025	26/30
Beck Depression Inventory (BDI)	February 20, 2025	12/60
Beck Anxiety Inventory (BAI)	February 20, 2025	9/63
Post-traumatic Stress Disorder Checklist for DSM-5 (PCL-5)	March 4, 2025	24/80
Automatic Thoughts Questionnaire (ATQ)	February 20, 2025	19/85

All psychological assessments in Table 2 were administered while the patient was experiencing dissociative amnesia, prior to the recovery of her memory of the incident. Consequently, the low psychometric scores on certain assessment tools like the BDI and PCL-5 likely reflect limited insight and lack of emotional awareness during this amnesic period rather than the absence of depressive or post-traumatic symptoms.

Treatment outcome

Based on the clinical findings, investigations, and observations during admission, a diagnosis of Major Depressive Disorder with Dissociative Amnesia was made. She was started on oral duloxetine 30 mg once daily (OD) and received supportive psychotherapy, which helped her gradually process her emotions and guilt. Over time, she experienced significant improvement in mood stability and emotional regulation.

Ordinarily, cases of this nature are subject to police charges and subsequent referral for forensic psychiatric evaluation. In this case, however, charges were dropped, and the police did not disclose the reason for this decision. The decision not to pursue legal action significantly influenced the course of management. Hence, the main focus of this case was treatment and rehabilitation rather than legal adjudication. In the absence of a criminal charge, she was not transferred to a forensic psychiatric unit, and management proceeded within the framework of general psychiatric services. Nevertheless, forensic psychiatric and neuropsychiatric input were still sought to ensure a comprehensive assessment. After two months of psychiatric hospitalization, she was deemed stable with a low risk of suicide or further harm and was subsequently discharged for outpatient follow-up.

Her son was discharged from the Women and Children’s Centre earlier than the patient. A child protection assessment was completed by the Department of Social Welfare. The decision was that the child would be cared for by his father and babysitter under close monitoring. After discharge, the patient was allowed supervised visits to the babysitter’s home accompanied by her husband. Observation during the visit showed appropriate warmth and interaction between the child and the patient. The child did not demonstrate fear or avoidance.

She continues to attend our psychiatric outpatient clinic and has shown gradual improvement in her depressive symptoms. Counselling and psychoeducation continue to be provided to the patient regarding relapse warning signs, treatment adherence, and the importance of continued supervision.

## Discussion

Clinical considerations

This case highlights the complex intersection between dissociative amnesia and a filicide attempt, emphasizing the importance of comprehensive psychiatric assessment and legal-ethical considerations in such a tragic event. Filicide, though relatively uncommon, carries profound psychological, forensic, and societal implications. It is defined as the act of a parent killing their own child and represents one of the most emotionally and morally charged categories of homicide [3]. Resnick’s typology remains a cornerstone in understanding filicide motivations. He categorized filicide into five subtypes: altruistic, acutely psychotic, unwanted child, child maltreatment, and spousal revenge filicide [3]. In this case, while initial intent was obscured by dissociative amnesia, the patient later described overwhelming distress and suicidal ideation, suggestive of an altruistic motive rooted in perceived hopelessness and depressive cognition. However, given the impulsive nature of the act, the exact motive could not be definitively determined.

Attempted filicide has a strong association with parental psychiatric illness [7]. Numerous studies have consistently demonstrated a disproportionate prevalence of mental illness among perpetrators of filicide. For instance, a systematic review by Flynn SM et al. reported that approximately 40% of perpetrators had a diagnosable mental disorder, most commonly mood and personality disorders [5]. This overrepresentation underscores the importance of recognizing psychiatric vulnerability and aligns with the illustrated patient’s eventual diagnosis of major depressive disorder. Interestingly, despite a lack of prior psychiatric history, she later retrospectively revealed depressive symptoms such as low mood, anhedonia, lethargy, worthlessness, and suicidal thoughts in the weeks preceding the event, alongside functional impairment at work and in caregiving. These features fulfilled the DSM-5 diagnostic criteria for Major Depressive Disorder. These findings underscore the risk of untreated or unrecognized mood disorders progressing to crisis-level behaviors, especially in individuals experiencing social or emotional isolation.

Dissociative amnesia is classified in the DSM-5 under dissociative disorders. It is characterized by the inability to recall important autobiographical information, usually of a traumatic or stressful nature, not attributable to ordinary forgetfulness [1]. Dissociative amnesia specifically refers to a functional memory impairment, often without detectable structural brain lesions or identifiable organic causes. Various pathophysiological theories have been proposed, including disruptions of the frontal executive system leading to memory retrieval deficits, as well as cultural contamination or iatrogenesis contributing to the memory gap [8]. On an important note, while dissociation can indeed be considered a defense mechanism to cope with overwhelming psychological trauma [9], comprehensive investigations should be undertaken to rule out possible organic conditions that could potentially lead to dissociative amnesia. In this case, extensive workup, including CT and MRI of the brain, autoimmune panels, and cognitive testing (MMSE and MoCA), yielded no abnormalities, thus reinforcing a functional etiology for the amnesia. None of the medical workups could account for her memory gap.

An intriguing dimension of this case is the gradual recovery of memory. To date, no evidence-based treatments exist for dissociative amnesia [8]. Some researchers propose that memory may be spontaneously retrieved when the individual is psychologically safe. In the ward, we primarily aimed to create a therapeutic space of emotional safety, free from coercion and judgment, which we believed could help promote spontaneous recovery of memory [10]. Approximately one month into her psychiatric admission, recovery of memory occurred, initially through distressing nightmares, eventually culminating in vivid recollections. The recovery of memory was accompanied by intense remorse and emotional distress. From a different angle, some researchers argue that external cues (e.g., repeated retelling by family or clinicians) might facilitate pseudo-recollection or confabulated narratives [8]. This poses a challenge in forensic psychiatry, namely, determining whether recollected memories are genuine or constructed. Nonetheless, the patient’s consistent emotional response, congruent narrative, and absence of secondary gain (e.g., legal advantage, given that police did not press charges) lend credence to the authenticity of her memory retrieval.

Differential diagnoses considered

At the time of admission, clinical evaluations such as the BDI and the PCL-5 were not indicative of active psychopathology. The negative findings from objective psychological assessments posed significant diagnostic challenges, particularly when a filicide-suicide occurred in the context of dissociative amnesia, which masked both the index offense and underlying psychiatric symptoms. The discrepancy between the clinical history and collateral information from the patient’s family, who reported early warning signs such as emotional distress, hopelessness, and caregiving-related stress, further complicated the assessment. Undoubtedly, formulating a diagnosis in this context is challenging, particularly when symptoms are masked, intent remains unclear, and the patient herself has no recollection of the event.

In the present case, the primary diagnosis upon discharge was major depressive disorder with dissociative amnesia. As elaborated earlier, the patient had exhibited core features of a major depressive episode preceding the event, including low mood, anhedonia, early-morning awakening, lethargy, worthlessness, suicidal ideation, and functional impairment. The above paragraphs outline how the patient’s symptoms fulfilled the diagnostic criteria for major depressive disorder and dissociative amnesia as described in the DSM-5. Of note, adjustment disorder with depressed mood was considered in view of an identifiable psychosocial stressor and initial subthreshold depressive symptoms. However, the eventual discovery of a preceding full major depressive episode with suicidal thoughts supported a diagnosis of major depressive disorder rather than adjustment disorder.

In the early stage of assessment, acute stress reaction was considered, as the patient experienced severe psychological trauma with a sudden onset of dissociative symptoms. According to the ICD-11, acute stress reaction typically arises within hours to days following exposure to a stressful event and begins to subside within a few days after the event [11]. While the patient did exhibit a stress response in the form of amnesia for the event, she lacked other core features of acute stress reaction such as autonomic hyperactivity. More importantly, the dissociative amnesia in this case persisted beyond what is typical in acute stress reaction, lasting for over one month during psychiatric admission. The recovery of memory, which began via nightmares approximately one month later, is inconsistent with the usual resolution timeline of acute stress reaction.

Dissociative identity disorder was also considered in the differential due to the presence of amnesia surrounding a traumatic incident. However, it was ruled out based on DSM-5 diagnostic criteria, which require the presence of two or more distinct personality states or marked discontinuity in a sense of self or agency, accompanied by related alterations in affect, behavior, consciousness, memory, perception, cognition, or sensory-motor functioning [1]. In this patient, there was no evidence of identity disruption, dissociative switching, or episodes of behavioral change observed by collateral sources. Across repeated clinical interviews, she consistently demonstrated a stable sense of self without fragmentation, third-person speech, or referential phrasing. Her amnesia was strictly circumscribed to a single, time-limited episode, i.e., the traumatic event itself, with intact autobiographical continuity before and after. There were no signs of pervasive dissociation, fugue states, or loss of agency. Thus, a diagnosis of dissociative identity disorder was not supported.

Moreover, catatonia was considered, given the patient’s initial emotional blunting and reduced speech. However, classic catatonic features such as stupor, negativism, posturing, and waxy flexibility were absent throughout her hospital stay [1]. The patient remained responsive, communicative (albeit guarded initially), and oriented. In addition, there were no signs suggestive of medical, neurological, or substance-related causes of catatonia. Her condition improved gradually with supportive psychotherapy and antidepressant treatment, further supporting an alternative diagnosis.

Another crucial consideration is malingering, where individuals feign memory loss for personal benefit. Although malingering is always a diagnostic possibility in forensic contexts, several features in this case argue against it. First, the patient had no prior legal or psychiatric history, and there was no apparent external gain, particularly after charges were dropped. Second, she demonstrated persistent, convincing amnesia for weeks despite being in a therapeutic environment, which would be difficult to sustain without inconsistencies. Third, structured interviews and testing (e.g., MMSE, MoCA) showed consistent performance without signs of exaggeration or deception, and her eventual distress upon recollection supports a genuine emotional struggle.

Legal and forensic aspects: a missed opportunity

Under the Malaysian Criminal Procedure Code (CPC), individuals accused of criminal offences and suspected to be of unsound mind are typically assessed under Section 342, following a court order [12]. If a judge or magistrate finds that an accused person is of unsound mind and consequently incapable of making a defence, the court may order that the accused be sent to a psychiatric hospital for assessment by forensic psychiatry specialists [12]. In the current case, no such evaluation occurred, as the legal authorities decided not to file charges, eliminating the judicial pathway for mandating assessment. This posed a significant limitation and a missed opportunity. In comparable cases where legal proceedings do occur, the assessment of mens rea (criminal intent) becomes critical in determining culpability. Had the patient been sent to a forensic psychiatric hospital, the evaluation would have incorporated more structured assessments tailored for legal proceedings. These may include detailed documentation of the patient’s mental state at the time of the offence, retrospective capacity and intent analysis, assessment of criminal responsibility, and risk formulation for future offending [13].

While the absence of a forensic pathway meant no formal court report was generated, the core elements of psychiatric assessment and risk management were still addressed in the general setting of this case. One of the central dilemmas involved determining the intentionality of the act, particularly during the period of amnesia. The absence of clear intent in the context of dissociative amnesia became a pivotal clinical challenge in determining whether the act was impulsive, premeditated, or psychotically driven. Intent is fundamental in distinguishing between deliberate homicide attempts and acts committed under impaired volition. The lack of recall, coupled with non-suggestive initial psychiatric findings, hindered the ability to ascertain mens rea. The literature has described how dissociative amnesia can profoundly affect a person’s criminal responsibility, particularly when insight is limited and emotional blunting masks psychological distress [14].

Although certain structured forensic tools were not employed in this case due to jurisdictional limitations, their inclusion in future similar cases could enhance the accuracy and objectivity of psychiatric assessments. This case also raises broader questions about inter-agency coordination and whether the threshold for legal referral in psychiatric emergencies is consistently applied. A standardised forensic evaluation framework may be beneficial even in cases without criminal charges, especially when outcomes carry high psychosocial or public safety implications.

## Conclusions

In conclusion, this case underscores the multifactorial complexity of a filicide-suicide attempt involving dissociative amnesia and a mood disorder. It illustrates the diagnostic challenges posed by memory disturbances, the importance of collateral information, and the forensic implications of amnesia in violent acts. Future encounters with similar cases may benefit from a structured, multidisciplinary approach integrating psychiatric, neurological, and forensic perspectives. The patient’s recovery also offers valuable insight into the potential for memory restoration and emotional processing in the presence of a supportive and non-judgemental therapeutic environment. The mind can fracture, even in the setting of unrecognised depressive symptoms, and that fracture can carry devastating consequences if left unseen.
